# Editorial: Auditory perception and phantom perception in brains, minds and machines

**DOI:** 10.3389/fnins.2023.1293552

**Published:** 2023-10-06

**Authors:** Achim Schilling, Roland Schaette, William Sedley, Richard Carl Gerum, Andreas Maier, Patrick Krauss

**Affiliations:** ^1^Neuroscience Lab, University Hospital Erlangen, Erlangen, Germany; ^2^Cognitive Computational Neuroscience Group, University Erlangen-Nürnberg, Erlangen, Germany; ^3^UCL Ear Institute, University College London, London, United Kingdom; ^4^Faculty of Medical Sciences, Newcastle University, Newcastle upon Tyne, United Kingdom; ^5^Department of Physics and Astronomy, York University, Toronto, ON, Canada; ^6^Pattern Recognition Lab, University Erlangen-Nürnberg, Erlangen, Germany

**Keywords:** tinnitus, deep neural networks, artificial intelligence, auditory neuroscience, auditory phantom perception, cognitive computational neuroscience, neuroimaging, hearing loss

## Background and aim

The idea of combining artificial intelligence, in particular deep neural networks, and computational modeling with neuroscience and cognitive sciences has recently gained popularity leading to a novel research philosophy (Kietzmann et al., 2017; Kriegeskorte and Douglas, 2018; Naselaris et al., 2018). Regarding the auditory system, this interdisciplinary approach and close cooperation with computational sciences, would open up many interesting opportunities for auditory neuroscience. In analogy to lesion studies, phantom perceptions may serve as a vehicle to understand the fundamental processing principles underlying auditory perception (Schilling et al., 2023). The prime example of an auditory phantom perception is subjective tinnitus, i.e., the perception of a sound without any physical sound source involved (Krauss et al., 2016). Impairments of the auditory system due to cochlear damage, in particular synaptopathy (Tziridis et al., 2021), can lead to subjective tinnitus. Approximately 12% of the population are affected by tinnitus and 2% suffer heavily from that phantom perception, which causes severe side-effects ranging from concentration difficulties up to depression and suicide (Cederroth et al., 2020). A recent study estimated that in Germany alone the annual socio-economic costs of tinnitus are approximately 20 billion euros, which is in the same order of magnitude as the socio-economic costs of diabetes (Tziridis et al., 2022). Unfortunately, the underlying mechanisms of tinnitus are not yet fully understood and thus the development of accurate therapy approaches is difficult. The fact that tinnitus is associated with several mal-adaptations of the brain in different brain regions ranging from the brainstem up to the auditory cortex, makes it difficult to identify the exact underlying mechanisms (see e.g., Schilling et al., 2023). However, novel tools in neuroscience as well as recent progress in artificial intelligence and computational science provide novel approaches to unravel the mysteries of impaired auditory processing in the brain.

The aim of this Research Topic was to bring together researchers from different scientific fields such as neuroscience, artificial intelligence, psychology, medicine, computational science, and cognitive science to generate a trans-disciplinary view on auditory phantom perception and to spread novel research and therapy approaches with researchers from all fields. In this Research Topic, 19 publications were submitted, from which 13 (68.4%) were accepted for publication, resulting in a rejection rate of 31.6%.

## Main

The 13 publications were grouped into four different sub-topics: (1) Mechanistic Models of (impaired) Auditory Perception and Phantom Perception and the influence of Cross-Modality, (2) The Cerebellar Forward Model, Prediction Errors, Auditory Cognition and Schizophrenia, (3) Artificial Intelligence as a Tool and a Model to Understand the Brain, (4) Artificial Intelligence to Validate and Improve Treatment of Disorders of the Auditory System.

### Mechanistic models of (impaired) auditory perception and phantom perception and the influence of cross-modality

In this paragraph, we summarize studies, which deal with the effects of cross-modal connections, attentional fluctuations, as well as circadian rhythms on the auditory system and the other way around (Yakunina and Nam; Wang et al.; Grimm et al.; Kondo et al.). Furthermore, different mechanistic tinnitus models such as central noise in central gain are discussed amongst others in the light of correlations of tinnitus and decreased cognitive decline in certain tinnitus populations (Chen F. et al.; Hamza and Zeng).

In their study, Wang et al. analyzed the different neural correlates of Idiopathic Sudden Sensorineural Hearing Loss (ISSNHL, 30 dB hearing loss at several frequencies) with and without vertigo. The authors applied resting state functional magnetic resonance imaging and analyzed the regional homogeneity (ReHo, a measure of neural synchrony in certain brain regions) and the functional connectivity between brain regions. The main findings were: that in ISSNHL with additional vertigo the ReHo was decreased especially in the ipsilateral auditory cortex. Furthermore, the functional connectivity within the inferior parietal gyrus—a multi-sensory area processing vestibular information—was increased. The authors speculate that the dysfunction of the inferior parietal cortex affects the auditory cortex and worsens auditory processing.

Kondo et al. investigated the attention mechanisms in the auditory system. In their study, the authors applied functional magnetic resonance imaging (fMRI) to investigate which brain dynamics lead to attentional fluctuations. Thus, the authors measured the reaction time and the BOLD (blood oxygenation level dependent) responses during an auditory gradual onset, continuous performance task (gradCPT), where subjects have to quickly distinguish between a male (go trial) and female (no go trial) narrator. The authors applied energy landscape analysis and could show that task-related activation patterns cluster in different attractors. Indeed, it is assumed that attention plays a critical role also in the manifestation and modulation of tinnitus (Roberts et al., 2013). Thus, the methods and findings of this study could be a further step to unravel the neural origin of tinnitus.

The focus of the following four studies lies on tinnitus mechanisms, cross-modal influence on tinnitus and the connection of tinnitus and cognitive decline.

Yakunina and Nam discuss in their study the applicability, safety, and effectiveness of transcutanious and standard invasive vagus nerve (tVNS respectively VNS) stimulation for the treatment of tinnitus. The authors state that although the VNS technique paired with sensory stimuli drives plasticity in the cortex, the exact neural mechanisms regarding neuromodulation through (t)VNS remain elusive. The authors are clearly critical of (t)VNS stimulation in combination with auditory stimuli as tinnitus therapy for several reasons. In particular, the authors state that the entire field of combined VNS and auditory stimulation is based on the work of a single research group and was not independently confirmed by author researchers. However, recently the positive effect of combined somato-sensory and auditory stimulation on tinnitus has been confirmed in a double-blind study with a sample size of 326 tinnitus patients (Conlon et al., 2020). Yakunina and Nam further criticize that the combined VNS and auditory stimulation technique is exclusively based on the view that the main neural correlate of tinnitus is a reorganization of the tonotopy of the auditory cortex. Indeed, this view has been refuted several times in the meantime (see e.g., Koops et al., 2020). Hence, the study of Yakunina and Nam illustrates that a mechanistic theory behind auditory phantom perception is needed to develop perfect-fit therapy approaches.

The meta-analysis of Chen F. et al. is a further study dealing with mechanistic tinnitus models, and especially the connection of tinnitus and hyperacusis. The authors statistically evaluated and summarized the effect of acoustic trauma on auditory brainstem responses and discuss their evaluations in the light of synaptopathy at the inner hair cells, hyperacusis and tinnitus. The authors report a strong evidence for reduced wave I amplitudes—indicating spiral ganglion activity—in tinnitus patients compared to control groups. Furthermore, the authors state that wave I amplitude decrease is a proxy for synapse loss. However, for wave V the results are contradictory. In the literature, a tinnitus related increase as well as decrease of wave V is reported. Thus, the authors refer to the publication of Knipper et al. (2020), and speculate that the increased wave V amplitudes might be related to hyperacusis and could be explained with the central gain model that postulates an increased sensitivity of neurons through homeostatic plasticity, whereas central noise as the potential cause of tinnitus [would not lead to increased wave V amplitudes (Zeng, 2013, 2020; Schilling et al., 2023)].

In their study on circadian sensitivity of noise trauma-induced hearing loss and tinnitus in Mon- golian gerbils, Grimm et al. discovered a connection of tinnitus and decreased hearing thresholds [which has already been proposed by Krauss et al. (2016)]. In particular, the authors showed that in gerbils the highest hearing loss in the more affected ear occurs when the noise trauma is applied at 5 p.m., when the male gerbils have their activity minimum. This is a counter-intuitive finding, as mice and other rodent species are more sensitive at night, where their activity level is maximal. Furthermore, the authors report that the correlation of hearing loss and effect size of the behavioral signs of tinnitus in rodents (compare e.g., Schilling et al., 2017) is significant, i.e., that tinnitus is correlated with better hearing. This finding is in line with the stochastic resonance model of auditory phantom perception (Krauss et al., 2016; Schilling et al., 2021).

Hamza and Zeng investigated the connection of higher brain functions and tinnitus in elderly people. They report that in a cohort of non-hispanic people aged between 60 and 69 years with a hearing loss of at least 25 dB, the presence of a tinnitus perception was associated with improved cognitive performance. The described effect is surprising in a sense that usually hearing loss is associated with impaired cognitive function, and hence tinnitus as a frequent comorbid condition of hearing loss was often suspected to also lead to impaired cognition. Earlier studies also described a negative effect of tinnitus on cognitive performance. However, the authors criticize that these studies did not control for confounding and interactive factors such as age. The authors speculate that tinnitus may have a benefit in a sense that tinnitus patients have less speech perception difficulties compared to patients suffering from hearing loss alone without tinnitus. This hypothesis is indeed in line with several recent studies (Schilling et al., 2021, 2022; Schilling and Krauss, 2022).

### The cerebellar forward model, prediction errors, auditory cognition and schizophrenia

The second section of this Research Topic focuses on schizophrenia and additional auditory verbal hallucinations (“hearing voices”) and consists of two studies that provide further insight into impaired multimodal integration and neural mechanisms in the cerebellum (Chen J. et al.; He et al.).

In their study, Chen J. et al. used resting state functional magnetic resonance imaging (fMRI) and arterial spin labeling to investigate cerebral blood flow and functional connectivity strength alterations in schizophrenia patients with verbal hallucinations compared to patients with- out verbal hallucinations and a healthy control group. “Hearing voices” or verbal hallucinations are characteristic symptom in schizophrenia. The authors report an increased CBF/FCS ratio (cerebral blood flow functional connectivity) in auditory perception and language processing areas of the cerebral cortex (left superior and middle temporal gyri) in the auditory verbal hallucinations group compared to the healthy control group. Further alterations are found in cerebellar structures. The authors speculate that the failure of the forward model of the cerebellum, which describes the cerebellum as a structure calculating discrepancies between sensory input and predictions (efferent copies) (Manto et al., 2012), may be a potential cause of auditory verbal hallucinations.

A further study on schizophrenia, where He et al. used the rubber hand illusion—i.e. the misinterpretation of a rubber hand as own hand induced by synchronous visual and tactile stimulation—to investigate differences in multi-sensory integration in schizophrenia patients with and without auditory verbal hallucinations. As schizophrenia patients are in many cases not able to identify the actual sources of their sensory perception, all schizophrenia patients (with and without auditory verbal hallucinations) showed an enhanced and earlier rubber hand illusion compared to a healthy control group. Surprisingly, patients with auditory verbal hallucinations experienced weaker illusions than the group with no auditory verbal illusions. However, when stimuli were presented asynchronously only in the group without auditory verbal hallucinations the rubber hand illusion decreased, which indicates that the impairment in temporal processing is increased in schizophrenia patients with auditory verbal hallucinations. The authors conclude that patients with auditory verbal hallucinations have multi-sensory processing dysfunctions and internal timing deficits.

### Artificial intelligence as a tool and a model to understand the brain

In the third paragraph of this editorial, two studies using modern AI approaches to extract information from neuroimaging data (fNIRS and EEG), in order to get a better understanding of (impaired) brain mechanism, are summarized (Yoo et al.; Li et al.).

Yoo et al. used Long-Short-Term-Memory (LSTM) networks to classify neural activity patterns in the auditory cortex, which were evoked by six different auditory stimuli. To measure the neural activity patterns the near infrared spectroscopy (fNIRS) was used, a technique based on near infrared light penetrating the head and thus used to measure oxygenation changes. The authors report that the LSTM networks achieve a better classification accuracy compared to a support vector machine (SVM), when the data of all 18 participants was used for training, whereas the SVM worked better, when each participant was regarded individually. Thus, the authors speculated that the poorer performance of the LSTM network was a result of too few training examples. However, the authors emphasized that for the LSTM networks no hand-crafted features were needed.

In contrast to the previously described study, Li et al. analyzed EEG data with machine learning techniques. In particular, the authors investigate how to apply machine learning techniques to EEG-connectivity features to estimate, whether the tinnitus is right/left-lateralized or binaurally perceived. The authors analyzed four different connectivity features and two time-frequency domain features, and applied four different machine learning techniques (two different support vector machines (SVM), a multi-layer perceptron (MLP), and a convolutional neural network) to these features. They conclude that bilateral tinnitus was characterized as altered connectivity in both auditory cortices, whereas left-lateralized tinnitus affected only the contra-lateral auditory cortex. Right-lateralized tinnitus, however, affects connectivity of the auditory cortices in both hemispheres.

Furthermore, the authors report tinnitus classification accuracies above 99 % for a SVM and MLP network. Potentially, these models could be used as objective method to determine tinnitus locations.

### Artificial intelligence to validate and improve treatment of disorders of the auditory system

In the fourth and last paragraph of this editorial we summarize studies, which have used AI approaches to validate and improve treatments of disorders of the auditory system.

Yoon investigates how the recognition of certain consonants can be improved by identifying and amplifying certain frequency and time ranges (target frequency and time ranges) of the presented consonants. The main finding of the study is that for conversationally produced (in contrast to artificial) consonants the removal of mutually disturbing frequency ranges (conflicting frequencies) has a negative effect on consonant recognition, whereas the amplification of the target frequency and time ranges is an efficient way to improve consonant recognition in healthy listeners. The author states that the findings presented in this study serve as the basis to develop an artificial intelligence (AI) based, individual fitting scheme for patients, who wear a hearing aid and a cochlear implant. This idea is in line with the call of Lesica et al. (2021) to exploit the novel opportunities of AI to improve the treatment of hearing impairments.

One further study analyzing medical treatment success using machine learning, is the study by Puga et al. where they analyze the differences in patient population properties at two German tinnitus centers (University Hospital Regensburg and Tinnitus Center-Charit'e Berlin). The authors report that the age distribution of patients in both tinnitus centers are significantly different from general German population and also slightly differ in both centers. Furthermore, the population of patients in Regensburg contains more males than the German population and the Berlin data set. The tinnitus questionnaire score is more similar in Berlin and Regensburg in the female population. The authors used machine learning techniques (linear regression, lasso, ridge, support vector regressor) trained on the Berlin data set to predict the treatment outcome in the Regensburg data set. In accordance with the finding that tinnitus questionnaire scores of females are more similar across tinnitus centers it was also easier to predict the treatment outcome in the female population than in the male population. The authors conclude that in future these analyses should be extended to other research centers, order to learn more about inter-and intra-center differences.

A further study combines AI algorithms with mobile health apps. Shahania et al. used data of 21 persons acquired with a mobile health app to investigate how to make better predictions on tinnitus perception and the effects on the patients. A contextual multi-armed bandit algorithm was used to answer the question, if it is advantageous to involve the data from all patients in specific predictions (global model) or if it is better to regard each patient separately or small groups of similar patients. The authors summarize that entity-centric models (each user regarded separately) are preferred, and stated that there is no guaranty that the multi-armed bandit converges to an optimal solution, which could be caused by the relatively small sample size.

## Concluding remarks

The 13 studies published in the Research Topic “*Auditory perception and phantom perception in brains, minds and machines*” demonstrate that an inter-disciplinary view on the auditory system is necessary to unravel mechanisms underlying healthy and impaired processing in this sensory system. The heterogeneity of the studies shows that widening the view to research on other sensory modalities such as the somato-sensory system, or even to other disciplines as AI and computational sciences can lead to significant progress in these research strands.

The fast growing field of AI and high-performance computing in combination with innovative trans-disciplinary ideas have the potential to further boost auditory neuroscience and audiology. Or as the famous neuroscientist Gershman (2023) states: “Happily, algorithms optimized for solving engineering problems frequently turn out to be successful models of brain function.”

Furthermore, the novel algorithms help to analyze and thus to transform neuro-imaging data into scientific knowledge on the one hand, and furthermore help to validate and improve therapy approaches for disorders of the auditory system such as tinnitus on the other hand. We hope that the interdisciplinary approach of this Research Topic inspires scientists of different fields to start co-operations in order to unravel the functions and mechanisms of the human brain.

## Author contributions

AS: Conceptualization, Funding acquisition, Project administration, Writing—original draft, Writing—review and editing. RS: Conceptualization, Project administration, Writing—review and editing. WS: Conceptualization, Project administration, Writing—review and editing. RG: Conceptualization, Project administration, Writing—review and editing. AM: Conceptualization, Project administration, Writing—review and editing. PK: Conceptualization, Writing—original draft, Writing—review and editing, Funding acquisition, Project administration.

## References

[B1] Cederroth C. R. Lugo A. Edvall N. K. Lazar A. Lopez-Escamez J. A. Bulla J.. (2020). Association between hyperacusis and tinnitus. J. Clin. Med. 9, 2412. 10.3390/jcm9082412 32731492 PMC7465629

[B2] Conlon B. Langguth B. Hamilton C. Hughes S. Meade E. Connor C. O.. (2020). Bimodal neuromodulation combining sound and tongue stimulation reduces tinnitus symptoms in a large randomized clinical study. Sci. Transl. Med. 12, eabb2830. 10.1126/scitranslmed.abb2830 33028707

[B3] Gershman S. J. (2023). What have we learned about artificial intelligence from studying the brain? Available online at: https://gershmanlab.com/pubs/NeuroAI_critique.pdf 10.1007/s00422-024-00983-2 38337064

[B4] Kietzmann T. C. McClure P. Kriegeskorte N. (2017). Deep neural networks in computational neuroscience. BioRxiv 4, 133504. 10.1101/133504 PMC5187453 28082889

[B5] Knipper M. Van Dijk P. Schulze H. Mazurek B. Krauss P. Scheper V.. (2020). The neural bases of tinnitus: lessons from deafness and cochlear implants. J. Neurosci. 40, 7190–7202. 10.1523/JNEUROSCI.1314-19.2020 32938634 PMC7534911

[B6] Koops E. A. Renken R. J. Lanting C. P. van Dijk P. (2020). Cortical tonotopic map changes in humans are larger in hearing loss than in additional tinnitus. J. Neurosci. 40, 3178–3185. 10.1523/JNEUROSCI.2083-19.2020 32193229 PMC7159897

[B7] Krauss P. Tziridis K. Metzner C. Schilling A. Hoppe U. Schulze H.. (2016). Stochastic resonance controlled upregulation of internal noise after hearing loss as a putative cause of tinnitus-related neuronal hyperactivity. Front. Neurosci. 10, 597. 10.3389/fnins.2016.00597 28082861 PMC5187388

[B8] Kriegeskorte N. Douglas P. K. (2018). Cognitive computational neuroscience. Nat. Neurosci. 21, 1148–1160. 10.1038/s41593-018-0210-5 30127428 PMC6706072

[B9] Lesica N. A. Mehta N. Manjaly J. G. Deng L. Wilson B. S. Zeng F. G.. (2021). Harnessing the power of artificial intelligence to transform hearing healthcare and research. Nat. Mach. Int. 3, 840–849. 10.1038/s42256-021-00394-z

[B10] Manto M. Bower J. M. Conforto A. B. Delgado-García J. M. Da Guarda M. Gerwig S. N. F.. (2012). Consensus paper: roles of the cerebellum in motor control—the diversity of ideas on cerebellar involvement in movement. The Cereb. 11, 457–487. 10.1007/s12311-011-0331-9 22161499 PMC4347949

[B11] Naselaris T. Bassett D. S. Fletcher A. K. Kording K. Kriegeskorte N. Nienborg H.. (2018). Cognitive computational neuroscience: a new conference for an emerging discipline. Trends Cognit. Sci. 22, 365–367. 10.1016/j.tics.2018.02.008 29500078 PMC5911192

[B12] Roberts L. E. Husain F. T. Eggermont J. J. (2013). Role of attention in the generation and modulation of tinnitus. Neurosci. Biobehav. Rev. 37, 1754–1773. 10.1016/j.neubiorev.2013.07.007 23876286

[B13] Schilling A. Gerum R. Metzner C. Maier A. Krauss P. (2022). Intrinsic noise improves speech recognition in a computational model of the auditory pathway. Front. Neurosci. 16, 908330. 10.3389/fnins.2022.908330 35757533 PMC9215117

[B14] Schilling A. Krauss P. (2022). Tinnitus is associated with improved cognitive performance and speech perception–can stochastic resonance explain? Front. Aging Neurosci. 14, 1073149. 10.3389/fnagi.2022.1073149 36589535 PMC9800600

[B15] Schilling A. Krauss P. Gerum R. Metzner C. Tziridis K. Schulze H.. (2017). A new statistical approach for the evaluation of gap-prepulse inhibition of the acoustic startle reflex (GPIAS) for tinnitus assessment. Front. Behav. Neurosci. 11, 198. 10.3389/fnbeh.2017.00198 29093668 PMC5651238

[B16] Schilling A. Sedley W. Gerum R. Metzner C. Tziridis K. Maier A.. (2023). Predictive coding and stochastic resonance as fundamental principles of auditory phantom perception. Brain 28, awad255. 10.1093/brain/awad255 37503725 PMC10690027

[B17] Schilling A. Tziridis K. Schulze H. Krauss P. (2021). The Stochastic Resonance model of auditory perception: a unified explanation of tinnitus development, Zwicker tone illusion, and residual inhibition. Prog. Brain Res. 262, 139–157. 10.1016/bs.pbr.2021.01.025 33931176

[B18] Tziridis K. Forster J. Buchheidt-Dörfler I. Krauss P. Schilling A. Wendler O.. (2021). Tinnitus development is associated with synaptopathy of inner hair cells in mongolian gerbils. Eur. J. Neurosci. 54, 4768–4780. 10.1111/ejn.15334 34061412

[B19] Tziridis K. Friedrich J. Brüeggemann P. Mazurek B. Schulze H. (2022). Estimation of tinnitus-related socioeconomic costs in Germany. Int. J. Environ. Res. Pub. Health 19, 10455. 10.3390/ijerph191610455 36012089 PMC9407899

[B20] Zeng F. G. (2013). An active loudness model suggesting tinnitus as increased central noise and hyperacusis as increased nonlinear gain. Hearing Res. 295, 172–179. 10.1016/j.heares.2012.05.009 22641191 PMC3593089

[B21] Zeng F. G. (2020). Tinnitus and hyperacusis: central noise, gain and variance. Curr. Opin. Physiol. 18, 123–129. 10.1016/j.cophys.2020.10.009 33299958 PMC7720792

